# Unveiling the multifaceted realm of human papillomavirus: a comprehensive exploration of biology, interactions, and advances in cancer management

**DOI:** 10.3389/fimmu.2024.1430544

**Published:** 2024-08-08

**Authors:** Meng Wu, Hui Huang, Ying Tang, Xuze Ren, Xinrui Jiang, Man Tian, Wei Li

**Affiliations:** ^1^ Department of Clinical Laboratory, Children’s Hospital of Nanjing Medical University, Nanjing, China; ^2^ Department of Rheumatology and Immunology, Children’s Hospital of Nanjing Medical University, Nanjing, China; ^3^ Department of Ultrasound Diagnostic, Children’s Hospital of Nanjing Medical University, Nanjing, China; ^4^ Department of Clinical Medicine, Clinical College of Anhui Medical University, Hefei, China; ^5^ Department of Neurology, Children’s Hospital of Nanjing Medical University, Nanjing, China; ^6^ Department of Respiratory, Children’s Hospital of Nanjing Medical University, Nanjing, China; ^7^ Department of Clinical Research, Children’s Hospital of Nanjing Medical University, Nanjing, Jiangsu, China

**Keywords:** HPV, HPV-associated cancers, HPV vaccines, microRNA, cancer prevention

## Abstract

Human Papillomavirus (HPV), an extensive family of DNA viruses, manifests as a persistent global health challenge. Persistent HPV infection is now firmly established as a significant aetiological factor for a spectrum of malignancies. In this review, we examine the latest insights into HPV biology and its intricate relationship with the host. We delve into the complex dynamics of co-infections involving HPV alongside other viruses, such as HIV, EBV, and HSV, as well as the burgeoning role of the microbiome in cancer development. We also explore recent advancements in understanding the specific contributions of HPV in the development of various cancers, encompassing cancers of the anogenital region, head and neck, as well as breast, lung, and prostate. Moreover, we focus on the current preventive strategies, including vaccination and screening methods, and therapeutic interventions that range from traditional approaches like surgery and chemotherapy to emerging modalities such as targeted therapies and immunotherapies. Additionally, we provide a forward-looking view on the future directions of HPV research, highlighting potential areas of exploration to further our understanding and management of HPV and its associated cancers. Collectively, this review is positioned to deepen readers’ understanding of HPV biology and its complex interplay with cancer biology. It presents innovative strategies for the prevention, management, and therapeutic intervention of HPV-associated malignancies.

## 1 Introduction

Cancer is a significant global health concern and constitutes an enormous social burden. Theories aiming to describe the tumorigenesis have been long thought to be connected (1). Viral infections have been recognized as important risk factors for the development of various types of cancer (2–4). Oncogenic viruses can induce cellular transformation through several mechanisms, including the integration of viral DNA into the host genome, expression of viral oncoproteins, and modulation of host immune responses (5). Moreover, viral infections play a pivotal role in the anoikis resistance within tumour cells, thereby enabling their evasion of programmed cell death and promoting the spread and metastasis of malignant cells (6).

Approximately 12% of human cancers are caused by viral oncogenesis (7). Among these viruses, HPVs are an established etiological agent of human cancer. Most HPV infections are asymptomatic and resolve spontaneously within a couple of years. However, some infections can persist and lead to the development of various diseases. It is estimated that HPV causes approximately 610,000 cancers cases (8) and more than 250,000 deaths each year worldwide (9). It also causes approximately 40,000 cases of head and neck (particularly oropharyngeal) cancers each year (10). Certain high-risk HPV types, particularly HPV-16 and HPV-18, are responsible for the majority of cervical cancer cases. Besides, these high-risk types can also cause other anogenital cancers, including anal, vulvar, vaginal, and penile cancers. HPV has been increasingly recognized as a cause of head and neck cancers, specifically oropharyngeal cancer (11). Additionally, HPV DNA was detected in a significant proportion of prostate cancer (PCa) tissue samples, with a positive rate of 32.7% (12). HPV infection was implicated in the development of PCa metastases, potentially by modulating the expression of genes associated with anoikis resistance (13). This finding underscores the potential role of HPV in the aetiology of PCa and warrants further investigation into the mechanisms by which HPV may contribute to the development and progression of the disease. In this context, prevention and early detection are key to reducing the burden of HPV-associated cancers. The available preventive measures include HPV vaccination, regular screening, and practicing safe sex. Vaccination against HPV is the most effective preventive measure. The HPV vaccine, originally developed to shield against cervical dysplasias, has demonstrated efficacy in countering a spectrum of anogenital malignancies linked to high-risk HPV types 16 and 18.

In this review, we will provide an overview of the structure, organization of HPV genome and concentrate on metabolic deregulations induced by the HPV viral oncoproteins. We will also summarize the mechanisms by which it induces cellular transformation and cancer development, and the strategies that can be employed for prevention and treatment. This review provides a broader perspective on HPV and its significant role in global cancer epidemiology.

## 2 Structure and organization of HPV genome

HPV is a small (50 to 60 nm in diameter and ~8 kb in length), non-enveloped, double-stranded DNA viruses (14). Currently, there are over 200 related variants of HPV that have been identified (15). These types are classified into five main genera, including alpha, beta, gamma, mu, and nu. Different types are associated with different disease and disease prevalence (16). The alpha genus infect both genital and oral mucosa, and can be associated with malignant disease, while the beta, gamma, mu, and nu genera target cutaneous that contribute to the formation of skin papillomas and skin warts (17). In addition, these types are often subdivided into low- and high-risk types based on their propensity to induce cancer. Among these, 14 types (HPV 16, 18, 31, 33, 35, 39, 45, 51, 52, 56, 58, 59, 66, and 68) are classified as high-risk due to their well-established association with different forms of cancer. These high-risk types, including HPV 16 and 18, are particularly notorious for their role in the pathogenesis of cancers such as cervical, oropharyngeal, anal, vulvar, vaginal, and penile cancers (18). In contrast, infection by low-risk HPV types, such as types 6, 11, 42, 43, and 44, is non carcinogenic and typically causes cutaneous genital lesions.

The genomic organization is highly conserved across the entire HPV family, contributing to the fundamental similarities in viral biology and pathogenesis among different HPV types. All HPV genomes are existed in infected cells as episomes. The icosahedral capsid is composed of 72 capsomers. HPV genome exists within the capsids, and are associated with cellular histones, forming chromatin-like structures.

Viral genomes harbors 8 open reading frames (ORFs) that is large enough to encode for a protein. The genome can be divided into three domains: an early gene coding region (E1, E2, E4, E5, E6 and E7), a late gene coding region (L1 and L2) and a long control region (LCR) (19).

**Figure 1:**
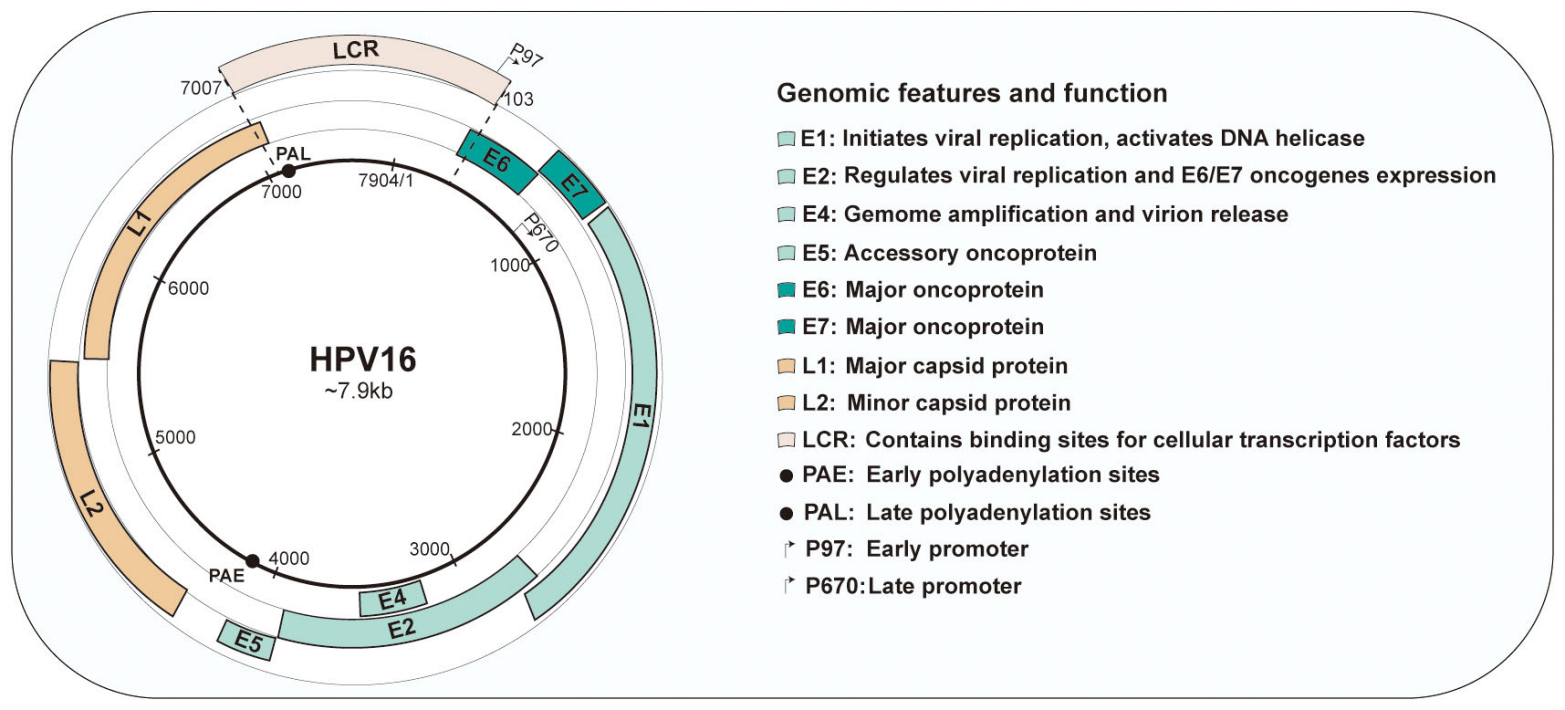
Genomic organization and functions of human papillomavirus type 16. HPV16 have approximately 8kb circular DNA genome containing eight open reading frames (ORFs) that are critical for different stages of the virus life cycle. The genome is divided into three main sections: the early region, containing the early genes(E1-E7); the late region, containing the late genes(L1and L2); and the long control region (LCR).

Of these viral proteins, E1 and E2 are participated in initiating viral DNA replication. E2 is thoughted to be the most vital protein in HPV genome because of its important role in viral transcription, replication, and genome partitioning. E2 also tightly regulates the expression of E6 and E7 oncogenes. Loss of E2 function contributes to increased expression of E6 and E7, further promoting cell proliferation and survival (20). E4 is considered to assist in in virus synthesis and release by disrupting cellular keratin in the upper epithelium (21). Occasionally, E5 functions as an accessory oncogenes that can augment the transforming activity of E6 and E7, contributing to tumor progression (22). E6 and E7 are oncoproteins that can alter host cell functions and cause the onset of cancer. Conversely, the late genes encode the L1 major and L2 minor structural capsid proteins, respectively, which expressed during the later stages of infection. These proteins are structural in nature and are responsible for forming the viral capsid. The LCR, localized between L1 and E6, is a non-coding, regulatory DNA region that plays a crucial role in the replication and transcription of HPV. It contains important regulatory elements that control the expression of viral genes and is involved in the initiation of DNA replication (23). Both promoters and enhancers play a critical role in regulating RNA transcription. Two major promoters reside in the genomes of HPV-16: an early promoter (p97) located within the LCR and a late promoter (p670) within E7. Furthermore, the early polyadenylation sites (PAE) is situated at the termination of E5 ORF, and the late polyadenylation sites (PAL) is located at the termination of L1 ORF. The genomic organization is presented in **Figure 1**.

Integrating the HPV genome into the host cell’s DNA is a pivotal step in HPV-associated carcinogenesis. HPV infects epithelial basal cells, pivotal for regeneration, and expresses the oncoproteins E6 and E7, which subvert cell cycle controls (24). Integration of HPV DNA into the host genome can inactivate tumour suppressors such as p53 and Rb, initiating uncontrolled proliferation (25). This event may evade immune surveillance and induce genomic instability, promoting carcinogenesis (26). The integration is a critical step in the progression to cervical and anogenital cancers, linked to advanced lesion grades and poorer prognosis (27).

## 3 Molecular mechanisms of HPV carcinogenesis

The carcinogenic potential of Human Papillomavirus (HPV) lies in its ability to integrate its DNA into the host cell genome and produce viral oncoproteins that interfere with normal cellular processes. HPV oncoproteins E6 and E7 are crucial in the initiation and progression of carcinogenesis. The primary identified roles of E6 and E7 is their abilities to degradation of the tumor suppressors proteins p53 and the retinoblastoma protein (pRb), respectively, leading to uncontrolled cell growth.

E6 is small protein of approximately 150 amino acids and contains two zinc binding domains (28). The E6 of high-risk HPV strains possesses multiple activities that promote the development of cancer. E6-associated protein (E6AP), which functions as an E3 ubiquitin protein ligase, is essential for the degradation of specific target proteins and assists in the carcinogenic characteristics of HPV. E6 binds the tumor suppressor protein p53 leading to its degradation via E6AP-mediated ubiquitination (29). However, E6 needs to bind to E6AP firstly demonstrate that neither E6 nor E6AP interacts significantly with p53 in the absence of the other (30). Thus, degradation of p53 results in the inactivation of p21, which known as cyclin-dependent kinase inhibitor, further preventing G1-S phase entry of the cell cycle (31). This allows cells with DNA damage or mutations to proliferate unchecked, increasing the risk of cancer development.

E6 is also recognized to impact different cellular factors within the host and interfere with host signaling pathways. HPV E6 protein interacts with FADD and caspase-8 to dysregulate the extrinsic apoptosis pathway, which transmits extracellular apoptotic signals from the cell surface (32). One mechanism by which HPV E6 protein inhibits BAX and BAK is through the promotion of their degradation, similar to p53 (33, 34). The degradation of BAX and BAK prevents their activation and impairs their ability to induce apoptosis. Additionally, E6 directly regulates the immune system by down-regulating interferon regulatory factor 3(IRF-3), diminishing the immune reaction to HPV antigens (35). PDZ domain-containing proteins are cellular proteins engaged in various cellular processes, including cell signaling and protein-protein interactions. HPV E6 has been found to interact with PDZ domain-containing proteins through a specific motif present in its C-terminal region known as the PDZ-binding motif. These interactions disrupt the normal function of these proteins, which are involved in cell signaling pathways and cell adhesion complexes, contributing to the oncogenic properties of HPV (36). E6 can also inactivate the transcriptional coactivator complex p300/CBP, thereby regulating the progression and differentiation of the cell cycle, and proteins involved in apoptosis (37). The unbalanced expression of E6 can also be induced expression of human telomerase reverse transcriptase (hTERT), thereby achieving cell immortalization (38).

**Figure 2:**
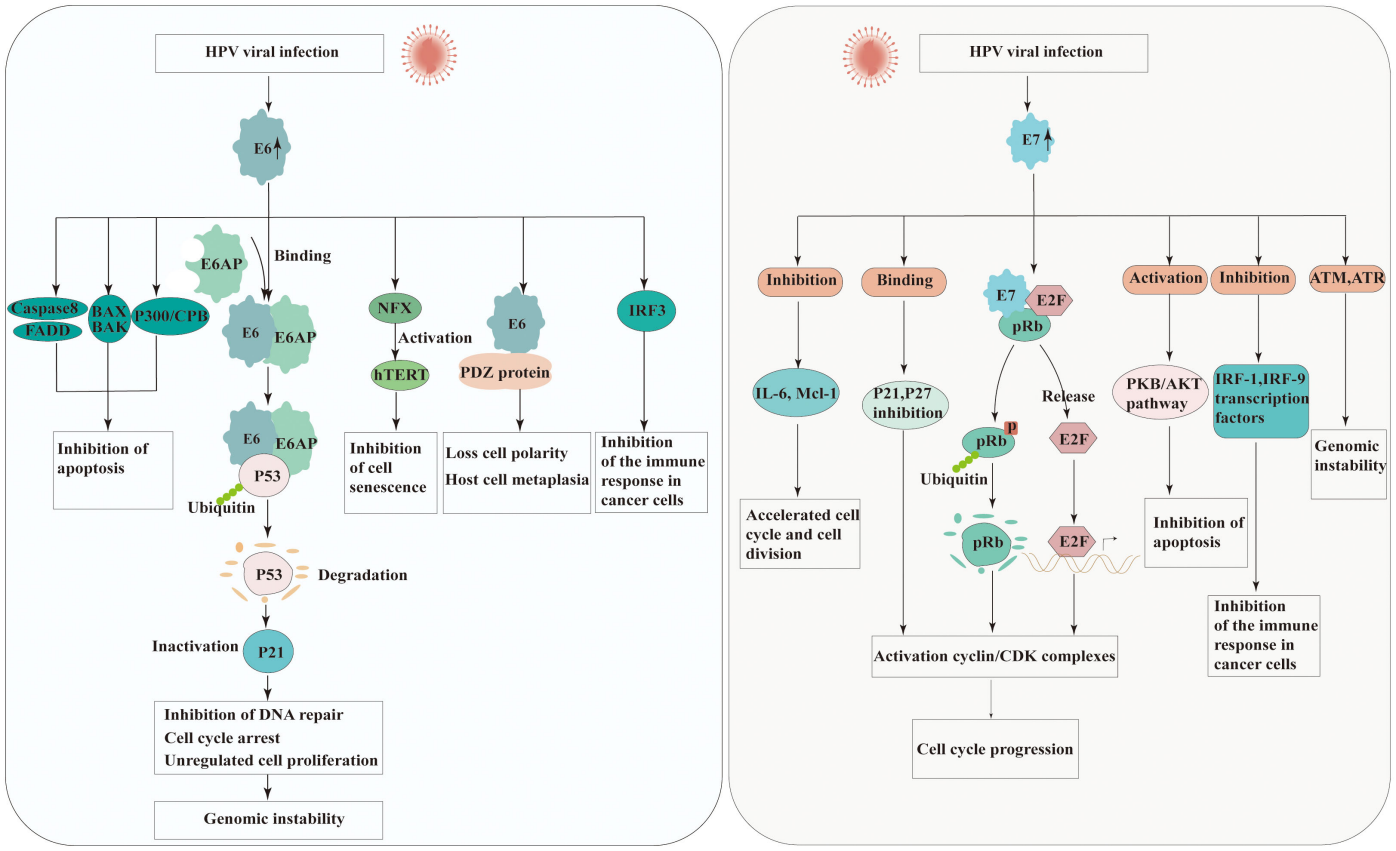
Molecular mechanisms of carcinogenesis by HPV E6 and E7 viral oncoproteins interacting with cellular protein. Left panel: the possible roles of HPV E6 in carcinogenesis. Right panel: the possible roles of HPV E7 in carcinogenesis. (E6AP, E6 associated protein; FADD, Fas-associated protein with death domain; BAK, Bcl-2 homologous antagonist/killer; BAX, Bcl-2-like protein 4; IRF, Interferon regulatory factors; NFX, X box-binding protein; hTERT, The TERT human protein; PDZ, Post-synaptic density proteins; CBP/300, CREB binding protein; pRb, Retinoblastoma protein; CDK, Cyclin-dependent kinase; IL-6, Interleukin 6; Mcl-1, Myeloid cell leukemia-1; PKB/AKT, Protein kinase B).

The E7 protein is approximately 100 amino acids in length and is characterized by the presence of conserved regions, including the CR1 and CR2 domains. CR-1 is required for transformation, whereas CR-2 contains the Rb binding domain. Similar to the E6 protein, the primary function of HPV E7 protein is to disrupt the cell cycle control mechanisms and promote cell proliferation. The E7 protein of high-risk HPV types primarily targets the pRb, another critical tumor suppressor protein. The interaction between E7 and pRb disrupts the Rb-E2F complex, leading to the release of E2F transcription factors. The released E2F factors then stimulate the transcription of genes involved in cell cycle progression, such as those encoding cyclins and cyclin-dependent kinases (CDKs). In addition to its interaction with pRb, HPV E7 protein also directly binds to and inhibits the activity of CDKs inhibitors such as p21 and p27, which normally function to inhibit cell cycle progression (39). By inhibiting CDKs, E7 further promotes cell cycle progression and and contributes to the development of HPV-associated cancers. One of the key mechanisms by which HPV E7 protein promotes carcinogenesis is through its interaction with the PKB/AKT pathway. HPV E7 protein enhances the activation of PKB/AKT by inhibiting PTEN, a known inhibitor of the PI3K (PI3 kinase) pathway. The activation of PKB/AKT promotes cell survival and proliferation, inhibits apoptosis, and contribute to the development and progression of HPV-associated cancers (40). Furthermore, E7 is also associated with a variety of other factors, such as Interleukin 6(IL-6) and Myeloid cell leukemia-1(Mcl-1), and causes the acceleration of cell cycle and cell division (40). Moreover, HPV E7 protein has mediated immune evading mechanism from IFN by binding to IRF-9 (41). The effect of HPV E6 and E7 in carcinogenesis is presented in **Figure 2**.

Additionally, the HPV E5 protein has also been reported to be involved in oncogenesis. One of its key functions is the activation of growth factor receptors, such as the epidermal growth factor receptor (EGFR). HPV E5 protein binds to EGFR and enhances its activation, resulting in continuous activation of downstream signaling pathways such as the mitogen-activated protein kinase (MAPK) and phosphatidylinositol 3-kinase (PI3K)/AKT pathways. This activation promotes cell proliferation, survival, and transformation, contributing to the development of tumor (42). E5 also disrupts the keratinocyte growth factor receptor signaling to inhibit autophagy and decrease the proliferation and differentiation of suprabasal keratinocytes (43). All these transformation events result in uncontrolled proliferation in tumor cells, which could be an initial stage in tumor development.

## 4 Coinfection and microbiome

In the context of HPV infection, coinfections with other viruses and alterations in the bacterial microbiome can significantly influence the course of the disease (44, 45). Herpesviridae and Polyomaviridae, detected in HPV-positive tumors, suggest a role for co-infection in disease progression and oncogenesis (46). These viruses can compromise the immune system’s ability to clear HPV, leading to a higher likelihood of persistent infection and subsequent progression to cancer. Nahand et al. reported that the HPV/EBV coinfection could be a significant contributing factor in the progression of prostate cancer. Furthermore, it highlights a potential role for EBV in facilitating the integration of the HPV genome, thereby influencing carcinogenesis (3).

Concurrently, alterations in the bacterial microbiome can also impact HPV infection and disease progression. The microbiome plays a pivotal role in HPV-driven carcinogenesis and cancers (47). Recent studies have suggested a potential link between vaginal microbiome dysbiosis and an increased risk of HPV persistence and cervical cancer development (48). It is plausible that the depletion of the microbiome composition by Lactobacillus may lead to a pro-inflammatory environment, thus potentially increasing malignant cell proliferation and the expression of HPV E6 and E7 oncogenes (49).

The interaction of HPV with coinfections and the microbiome is a critical research frontier, potentially enhancing our understanding of disease progression and informing new preventive and therapeutic HPV strategies.

## 5 The burden of HPV and HPV-associated cancers

The prevalence and incidence of HPV-associated cancers are influenced by various factors including sexual behavior, immune status (such as HIV co-infection), smoking, and access to screening and vaccination services. The burden of HPV is not evenly distributed globally (50). Despite the availability of effective preventive measures, the burden of HPV remains high. This is due to several factors including low vaccine coverage in some regions, lack of regular cancer screening, and gaps in public awareness about HPV and its associated risks (51). Regions with the highest rates of HPV include Sub-Saharan Africa, Latin America, and the Caribbean (26). In addition, the most profound burden of HPV lies in its strong association with various forms of cancer (52). HPV-associated cancers often have long latency periods, which means that the impacts of these diseases are not only immediate but also long-term, affecting individuals’ quality of life and survival rates. The treatment of these cancers can be complex and costly, placing a significant economic burden on healthcare systems. Apart from cancers, HPV also causes non-malignant conditions such as genital warts and recurrent respiratory papillomatosis (53). While not life-threatening, these conditions can cause significant psychosocial distress, further contributing to the overall burden of HPV.

## 6 Cancers caused by HPV

### 6.1 Head and neck squamous cell carcinomas

Head and neck squamous cell carcinomas (HNSCCs) are a diverse group of malignancies that arise from the mucosa lining the oral cavity, nasal cavity, larynx, hypopharynx, and oropharynx. These include oral squamous cell carcinomas (OSCC), oropharyngeal squamous cell carcinomas (OPSCC), laryngeal squamous cell carcinomas (LSCC) and nasal squamous cell carcinomas (NSCC). HPVs are responsible for a higher proportion of cases of OPSCC (33.6%) globally, while causing fewer cases of OSCC (22.2%) and LSCC (20.2%) (50). More than 90% of HPV-positive HNSCCs are mainly attributable to a single HPV type, HPV16 (54). In recent years, developed countries have seen a decrease in HPV prevalence due to the introduction of vaccination programs. However, HNSCC has now surpassed cervical cancer as the most common HPV related malignancy in these countries (10). Over the past few decades, there has been a significant increase in the incidence of OPSCCs, linked to HPV, particularly HPV type 16 in many developed countries (55). About 70% of OPSCCs are linked to HPV, this type of cancer is more common in men. Traditional risk factors for OPSCCs include tobacco use and heavy alcohol consumption, HPV-positive OPSCCs are often diagnosed in younger individuals with little to no history of tobacco or alcohol use. Instead, sexual behaviors, including early age at first sexual intercourse and a high number of sexual partners, particularly oral sex partners, have been linked to an increased risk of HPV-positive OPSCCs.

HPV-positive OPSCCs are often distinct from other head and neck cancers in terms of their risk factors, symptoms, progression, and response to treatment (56). HPV-positive OPSCCs often presents with a lump in the neck, which is a swollen lymph node containing metastatic cancer. Other symptoms can include persistent sore throat, difficulty swallowing, and changes in the voice. In comparison with HPV- negative HNSCCs, HPV-positive HNSCCs is easier to metastasize, and its mechanism has not yet been fully elucidated. Moreover, HPV-positive cancers tend to have a better prognosis and respond better to treatment than HPV-negative counterpart.

Since HPV-positive HNSCCs does not have an effective vaccine to prevent it like cervical cancer, early-stage detection and diagnosis of HPV-positive HNSCCs are very important. HPV detection by several methods (HPV16 E1 PCR, HPV RNA sequencing, HPV DNA sequencing, et al.) is widely used in determining HPV status (57–61). However, the methods and strategies of HPV detection have not been clearly stipulated, and approach for determining HPV status often differences among various studies (62). In addition, several studies have identified body fluids based biomarkers for HNSCCs. These biomarkers are proposed as possible novel diagnostic and prognostic strategy. Detection of serum HPV16 E6 and E7 antibodies are more clinically relevant in OPSCCs. A recent review and meta-analysis investigated that HPV16 E6 antibody is a highly sensitive and specific biomarker for the detection of HPV-related OPSCCs at diagnosis (63). In addition, a growing body of clinical evidence has suggested that pretreatment circulating neutrophil count (CNC), circulating monocyte count (CMC), and circulating lymphocyte count (CLC) can be used as early prognostic markers for HPV- related and HPV-unrelated OPC (64). In some studies, higher pretreatment concentrations of CD8 T cells predicted a response to induction chemotherapy in HPV16 positive OPC patients (65).

MicroRNA (miRNA) is a small non-coding RNA molecule that plays a vital role in regulating gene expression at the post-transcriptional level. Deregulation of miRNAs have been proposed as key players in numerous human malignancies, including HNSCCs. Extracellular miRNAs can be measured in many kinds of body fluids, including serum, plasma, and saliva, making it possible to act as minimally invasive biomarkers. The expression of miRNAs are difference between HPV-positive HNSCCs and HPV-negative HNSCCs. A number of studies have elucidated the association between HPV/p16 status and miRNA signatures. Furthermore, miRNAs level has been highly involved in HPV-positive HNSCC pathogenesis, immune response, invasion, chemoresistant or radioresistant phenotypes. Numerous studies have examined causal links between specific miRNAs and HPV-positive HNSCCs development or progression. Some of the most commonly up-regulated miRNAs in HPV-positive HNSCC tumors are miR-9, miR-127, miR-196, miR-222, miR-455 (66), while the down-regulated miRNAs are miR-122, miR-124, miR-146a (67). **Table 1** summarizes body fluids based biomarkers in HPV-positive HNSCCs, including extracellular miRNA implicated as accurate biomarkers in HPV-positive HNSCCs. However, miRNA as a diagnostic biomarker has not been used as clinical practice to date. It’s important to note that the regulation between miRNAs and their target genes can be complex in different tumor microenvironments. Large-scale clinical practice and validation still required. A better understanding of the mechanisms could help clinicians choose appropriate HPV-positive HNSCCs treatments.

**Table 1:** Body fluids based biomarkers in HPV-positive HNSCCs.

Biomarkers	HNSCCs subtype	Sample Material	Clinical aspect	References
HPV DNA	HNSCCs , OPSCCs	Saliva, gargles	Diagnosis, recurrence	(54)
CtHPV DNA	OPSCCs	plasma	Diagnosis, prognosis, recurrence	(54, 57–61),
E6/E7 antibodies	OPSCCs, OPC	serum	Diagnosis	(54, 63)
CNC/ CMC/ CLC	OPC	peripheral blood	Prognosis	(64)
CD8^+^T cell	OPC	peripheral blood	Prognosis	(65)
Up-regulated microRNAs ( miR-9, miR-127, miR-196, miR-222, miR-455)	HNSCCs	saliva	Diagnosis	(66)
Down-regulated microRNAs (miR-122, miR-124, miR-146a)	HNSCCs	saliva	Diagnosis	(67)

CNC, circulating neutrophil count; CMC, circulating monocyte count; CLC, circulating lymphocyte count; OPC, oropharyngeal cancer; OPSCC, oropharyngeal squamous cell carcinoma.

### 6.2 HPV-associated anogenital cancers

Cervical cancer continues to pose a significant public health challenge, particularly in settings with limited resources where screening and preventive measures may be less accessible. The incidence of other anogenital cancers, such as those of the anus, penis, vagina, and vulva, while comparatively rare, is exhibiting an upward trend.It is now clear that HPV plays a pivotal role in the etiology of cervical cancer and its precursor, cervical intraepithelial neoplasia (CIN) (68). High-risk HPV types, particularly HPV 16 and 18, are found in the majority of all high-grade CIN and invasive cervical cancers. The development of vaccines against HPV has been a milestone in the prevention of cervical cancer (69, 70). These vaccines are highly effective in preventing persistent infections with HPV and the development of CIN.

Studies have shown that over 90% of anal cancers are associated with HPV, making it the most significant risk factor for this type of cancer (71). HPV 16 has been found to be the most prevalent type in anal cancer. HPV is identified as a causative agent in approximately 25% of vulvar cancers (72), predominantly in younger women and particularly those with the high-grade squamous intraepithelial lesion, vulvar intraepithelial neoplasia (VIN). The majority of HPV-related vulvar cancers are associated with HPV type 16. Vaginal cancer is rarer than vulvar cancer, with HPV implicated in about 78% of cases (72). Similar to cervical and vulvar cancers, the primary high-risk HPV type associated with vaginal cancer is HPV 16.

HPV is a sexually transmitted infection that can infect the skin and mucous membranes, including those of the penis. It has been estimated that HPV is present in approximately 40-50% of penile cancers (73), with HPV 16 being the most commonly detected type. The virus can cause changes in the cells of the penis that may lead to penile intraepithelial neoplasia (PeIN), a precancerous condition, and eventually progress to invasive penile cancer. The majority of PeIN lesions are HPV positive, and they predominantly contain several HPV genotypes (HPV6, HPV11, or HPV16). A 2019 meta-analysis estimated that 98.6% of PeIN 1–2 lesions and 80.5% of PeIN 3 lesions were HPV positive (74). Risk factors for HPV-related penile cancer include a high number of sexual partners, history of genital warts, immunosuppression, smoking, poor personal hygiene, and uncircumcised status. Uncircumcised men are at a higher risk because the environment under the foreskin can favor HPV infection and persistence.

### 6.3 HPV in specific cancers (breast, lung, and prostate cancer)

HPV’s role in anogenital, head and neck, and cervical cancer is established, yet its association with other malignancies, such as breast, lung, and prostate cancers, is controversial and under investigation. Breast cancer(BCa) is the second most prevalent cancer globally, posing a significant health challenge, particularly in developed countries. Mortality rates for breast cancer among women significantly exceed those attributable to lung and colorectal cancers (75). Evidence linking HPV to BCa was first reported in 1992, sparking ongoing research into the potential role of the virus in the etiology of this malignancy. International studies, spanning Italy, Iran, Qatar, USA, Poland, Congo, Sudan, Ethiopia, Egyptian, Lebanese, Peruvian, Brazil, Mexico, South Africa and Ahvaz, have consistently reported the presence of HPV in women diagnosed with BCa (4, 76–96). The prevalence of HPV infection in BCa exhibits significant variability across studies over the past five years, with reported frequencies ranging from 2.7% to 77.2%(**Table 2**). Additionally, inconsistencies in HPV infection rates have been noted among different molecular subtypes of BCa, suggesting potential differences in susceptibility or etiological mechanisms (109). Globally, HPV types 16, 18, and 33 are the most prevalent, accounting for approximately 70% of all HPV-associated BCa cases (108). Additionally, other HPV strains, such as HPV 31, 39, 45, 52, and 58, have been identified in BCa samples, contributing to the diversity of the viral presence in these malignancies. On the other hand, Oliveira et al.’s research (97) found no HPV DNA in BCa tissues, prompting further inquiry into HPV’s role in carcinogenesis.

**Table 2:** Recent Studies about the role of HPV in Breast, Lung, and Prostate Cancers (2019–2024).

Country (Publication year)	Tumor type	Sample Source	Sample Size	HPV Positivity	Method	HPV Types Detected	References
Italy (2019)	BCa	FFPE	273	30.4%	CISH, PCR, NGS	16, 18 as the most prevalent	(76)
Iran (2019)	BCa	Fresh-frozen tissue	72	48.6%	PCR	16, 18, 33, 6, 11	(4)
USA (2019)	BCa	FFPE	18	44.4%	PCR	11, 39 as the most prevalent	(78)
Qatar (2020)	BCa	Fresh-frozen tissue	50	10%	PCR-12 hrHPV types	16, 35, 58	(77)
Poland (2021)	BCa	FFPE	383	4.4%	PCR-21 HPV types	16	(79)
Congo (2021)	BCa	FFPE	40	15%	PCR-14 hrHPV types	16 as the most prevalent	(80)
Sudan (2021)	BCa	FFPE	150	8.7%	PCR	16, 58, 18, 11	(81)
Ethiopia (2021)	BCa	FFPE	75	2.7%	PCR-19hrHPVs, 9lrHPVs	16, 6	(91)
Iran (2021)	BCa	FFPE	59	11.8%	PCR	18, 6	(83)
USA (2021)	BCa	FFPE	90	21.1%(HPV 6,11), 43.3% (HPV 16, 18)	CISH–HPV 6, 11, 18, 18	16, 18, 6, 11	(84)
Qatar (2021)	TNBC	FFPE	70	53%	PCR-14 hrHPV types	52, 45, 31, 58, 68	(85)
Egyptian (2021)	BCa	FFPE	40	17.5%	PCR	n.a.	(86)
Egyptian (2021)	BCa	Fresh-frozen tissue	40	50%	PCR	n.a.	(86)
Lebanese (2021)	BCa	FFPE	102	65%	PCR–14 hrHPV types, TMA	52, 35, 58, 45, 16 and 51 as the most prevalent	(87)
Iran (2021)	BCa	Fresh-frozen tissue	36	33.3%	PCR	16, 18, 31, 6	(88)
Egyptian (2021)	BCa	Fresh-frozen tissue	72	22.2%	PCR-HPV 16, 18, 31	16, 18	(89)
Peruvian (2021)	BCa	Fresh-frozen tissue	447	2.9%	PCR	16, 18	(90)
Iran (2022)	BCa	FFPE	63	17.89%	PCR	11, 16, 31, 33	(82)
Brazil (2022)	BCa	FFPE	75	0%	PCR	n.a.	(97)
Qatar (2022)	BCa	FFPE	74	65%	PCR-11 hrHPV types	n.a.	(92)
Mexico (2022)	BCa	FFPE	59	20.3%	PCR-32 hrHPV and lrHPV types	42, 31, 59 as the most prevalent	(93)
South Africa (2023)	BCa	FFPE	101	77.2%	PCR	16, 51, 70, 35 and 82	(94)
Iran (2023)	BCa	Fresh tissue	40	22.5%	PCR	18	(95)
Ahvaz (2023)	BCa	FFPE	100	7%	PCR	16	(96)
Brazil (2019)	AdC, SQC	FFPE	77	0%	Multiplex PCR, RT-PCR	n.a.	(98)
China (2020)	NSLC, SCLC	Fresh-frozen tissue	140	9.3%	PCR, Reverse hybridization	16, 18, 42, 44,51, 35/42/44	(99)
Iran (2020)	AdC, SQC, SCLC	Fresh-frozen tissue	102	52.9%	PCR, INNO-LiPA	16 as the most prevalent	(100)
Czech (2020)	AdC, SQC, LCC	FFPE	80	0%	qPCR	n.a.	(101)
China (2021)	SCLC	TBNA cells	310	59%	qRT-PCR	16	(102)
Iran (2021)	AdC, SQC, SCLC	Fresh-frozen tissue	109	51.4%	PCR, INNO-LiPA	16 as the most prevalent	(2)
China (2021)	AdC, SQC	Peripheral blood	100	16%	PCR	16	(103)
Spain (2022)	AdC, SQC, NSLC, SCLC	FFPE	41	56%	RT-PCR	16, 18, 33, 56, 58	(104)
Iran (2020)	PCa	Fresh-frozen tissue	58	32.7%	PCR, INNO-LiPA HPV Genotyping Kit	2, 6, 16, 18, 33	(12)
Iran (2021)	PCa	Fresh-frozen tissue	72	36%	qPCR	2, 6, 11, 16, 18, 33	(13)
Iran (2022)	PCa	Fresh-frozen tissue	73	28.7%	PCR	6, 11, 16, 18, 33	(105)
Morocco (2022)	PCa	Fresh-frozen tissue	50	16%	PCR	18	(106)
UK (2023)	PCa, Benign controls	Fresh tissue	49	32.7%	HPV-HCR Genotype-Eph kit	16, 33, 35, 45, 52, 56, 58	(107)
China (2023)	PCa	FFPE	143	7%	Cobas 4800 HPV Test, DR HPV Genotyping IVD Kit	18, 52,53,62	(108)

BCa, Breast Cancer; FFPE, Formalin Fixed Paraffin Embedded; CISH, Chromogenic In Situ.

Hybdridization; NGS, Next Generation Sequencing; PCR, Polymerase Chain Reaction; TNBC, Triple Negative Breast Cancer; hrHPV, high-risk Human Papillomavirus; lrHPV, low-risk Human Papillomavirus; n.a., not applicable/not available; LCa, Lung cancer; TBNA, transbronchial needle aspiration; SCLC, Small Cell Lung Cancer; LUSC, Lung Squamous Cell Carcinoma; LUAD, Lung Adenocarcinoma; NSLC, Non-small cell lung Cancer; AdC, Adenocarcinoma;SQC, Squamous Cell Carcinoma; PCa, prostate cancer;UK, United Kingdom; PCR, polymerase chain reaction.

Lung cancer(LCa) remains a leading cause of cancer-related mortality globally, with its aggressive nature and late diagnoses contributing to high fatality rates. LCa is primarily categorized into small-cell lung cancer(SCLC)and non-small-cell lung cancer (NSCLC), with SCLC constituting about 20% and NSCLC approximately 80% of cases. Globally, HPV has been identified in a significant proportion of LCa cases, with approximately 31% of these cases testing positive for the virus (110). Since Syrjanen’s 1979 proposal linking HPV to lung carcinogenesis (111), extensive research has explored potential mechanisms, reflecting ongoing global scientific inquiry. The HPV oncoprotein interacts with pRb, triggering E2F release and promoting cell cycle progression in infected lung cells. Evidence has consistently implicated HPV infection in a substantial proportion of LCa cases across various geographical regions, underscoring its potential role in the etiology and progression of the disease (100, 102, 103). The HPV types, including HPV16, HPV18, HPV33, HPV42, HPV44, HPV51, HPV56,and HPV58, have been identified in lung tissue samples (2, 99, 103, 104). However, in a study conducted in the Czech Republic, Jaworek et al. reported the absence of HPV in 80 primary NSCLCs (101). Similarly, Silva et al. observed the lack of HPV in 77 NSCLC patients (98).

Prostate cancer (PCa) is the most frequently diagnosed solid malignancy in men and is a leading cause of cancer-related mortality worldwide (112). The presence of HPV in prostate tissue was initially identified by McNicol et al. through the application of PCR method (113), marking a pivotal discovery in the study of HPV’s potential role in prostate cancer. High-risk HPV types 16 and 18 are implicated in the immortalisation and transformation of normal prostate cells into malignant counterparts (114, 115). A comprehensive review on the association between PCa and HPV encompassed 60 studies, highlighting diverse methodologies such as PCR, ELISA, hybridization, and IHC. Of these, 51 focused on HPV detection in tissue samples, while 13 examined blood samples. Notably, 11 studies, representing 18% of the total, demonstrated a positive correlation between HPV presence and the development of PCa (116). In contrast to other findings, a study by Khoury et al. (117), employing Next-Generation Sequencing (NGS) on data from The Cancer Genome Atlas (TCGA), reported the absence of HPV in 53 cases of PCa.

**Figure 3:**
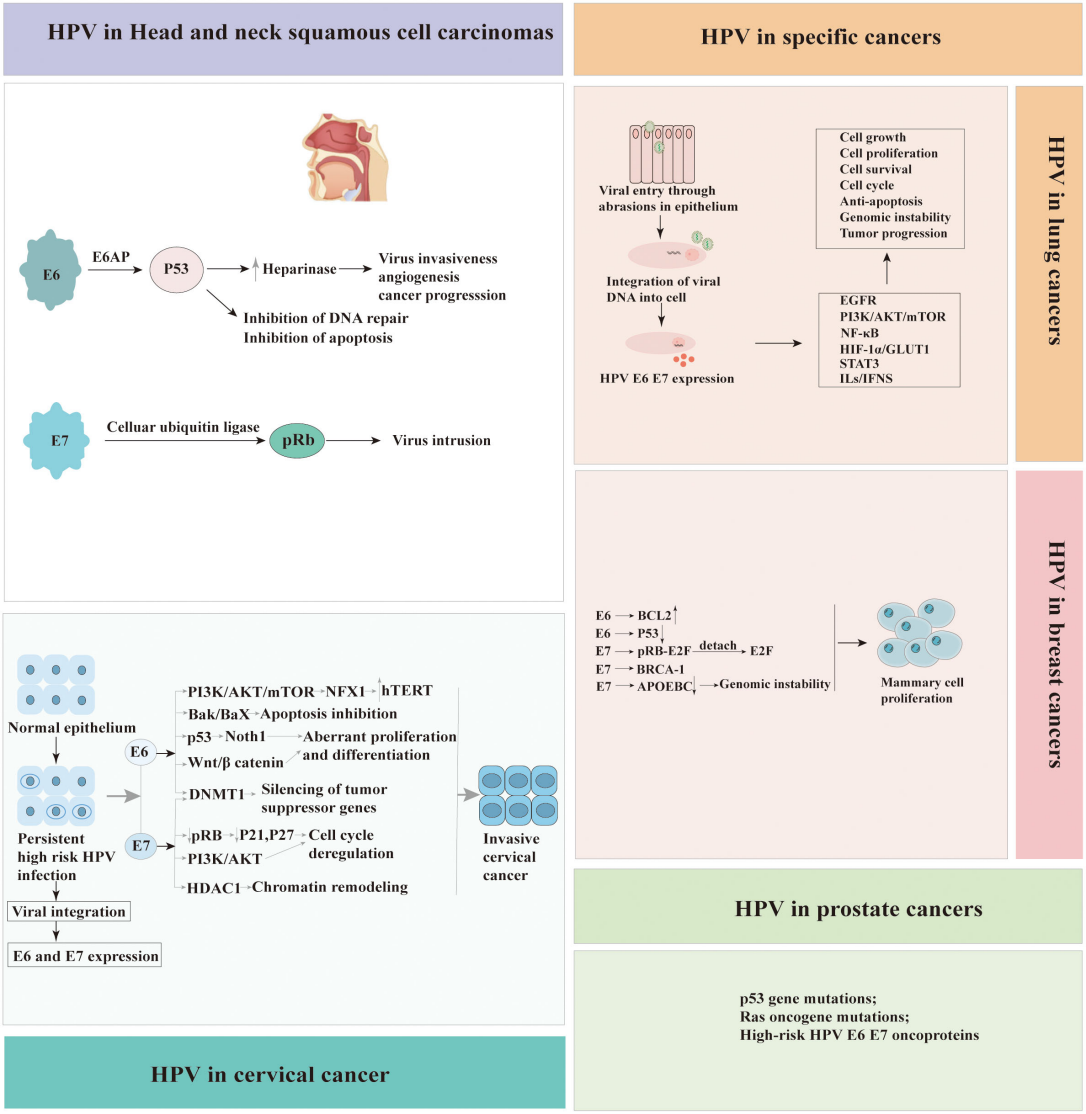
Pathogenic mechanisms of human papillomavirus (HPV)‐associated cancers.

In conclusion, variations in study design and subject characteristics, such as sexual behaviour, racial genetic predispositions, sample sizes, and methodological approaches, likely contribute to the inconsistent findings on the relationship between HPV infection and the development of BCa, LCa and PCa. To elucidate the role of HPV in these specific cancers, there is a necessity for comprehensive research employing larger sample sizes and standardized diagnostic methodologies. Recent studies about the frequency of HPV detection globally in BCa, LCa and PCa is presented in **Table 2** (105–107). Pathogenic mechanisms of HPV-associated cancers were summarized in **Figure 3**.

## 7 HPV-associated cancer prevention

Prevention and early detection are key to reducing the burden of HPV-associated cancers. The available preventive measures include HPV vaccination, regular screening, and practicing safe sex.

### 7.1 HPV vaccination

Vaccination against HPV is the most effective preventive measure (118). Vaccination for both males and females are recommended to protect against HPV-related cancers and genital warts. Vaccination typically begins at age 11 or 12, with catch-up vaccinations for older individuals. Vaccination of individuals up to age 26, and sometimes even older, may be recommended depending on local guidelines. The vaccine also produces a higher immune response in preteens than in older adolescents.

There are several HPV vaccines available worldwide that protect against both high risk HPV types that can lead to cervical cancer and low risk types that cause genital warts. In the last decades, five vaccines are currently licensed. These vaccines are based on virus like particles (VLPs), which self-assemble from the major capsid HPV L1 protein. Gardasil is the original Gardasil vaccine. It protects against four types of HPV: the two types that cause most cervical cancers (16 and 18) and the two types that cause genital warts (6 and 11). Gardasil 9 is the most widely used HPV vaccine, and it protects against nine types of HPV (6, 11, 16, 18, 31, 33, 45, 52, and 58) (119). These include the two types that cause 70% of cervical cancers (16 and 18) and five other high-risk types (31, 33, 45, 52, 58), as well as two types that cause 90% of genital warts (6 and 11). Cervarix protects against HPV types 16 and 18 only (120). Unlike Gardasil, it does not protect against the HPV types that cause genital warts. It is not used as commonly as Gardasil, but is still used in some countries. Even though the new (9-valent) vaccine seems promising, next-generation vaccines as well as awareness programs associated with HPV vaccination and budget reinforcements for immunization are needed (43). Cecolin was licensed in China in 2020 and prequalified by the World Health Organization (WHO) in 2021 (121). More recently, a recombinant bivalent vaccine, targeting HPV-16 and HPV-18, developed by Shanghai Zerun Biotechnology, was also licensed in China in 2022 and prequalified by WHO in 2022 (122). The main features of currently licensed prophylactic HPV vaccines was listed in **Table 3**. In addition, emerging evidence suggests that HPV vaccines might also provide protection against oral HPV infections that could lead to oropharyngeal cancers (123). However, there is no clear evidence for licensed prophylactic HPV vaccines in the prevention of OPSCC as there are no identified precursor lesions that could be used as efficacy endpoint for OPSCCs (124). More research is needed to confirm this potential benefit.

**Table 3:** Main features of currently licensed prophylactic HPV vaccines.

Vaccine	HPV strains targeted	Type of vaccine	Manufacturer and Date licensed	Adjuvant	Expression
Gardasil	HPV 6/11/16/18	Quadrivalent	Merck 2006	AAHS	Yeast
Gardasil 9	HPV 3/11/16/18/31/33/45/52/58	Nonavalent	Merck 2014	AAHS	Yeast
Cervarix	HPV 16/18	Bivalent	Glaxo SmithKline 2007	AS04	Baculovirus-insect cell
Cecolin	HPV 16/18	Bivalent	Xiamen Innovax Biotech 2020	Aluminum hydroxide	Escherichia Coli
Walvax recombinant HPV vaccine	HPV 16/18	Bivalent	Shanghai Zerun Biotechnology 2022	Aluminium phosphate	Yeast

AAHS, Amorphous aluminum hydroxyphosphate sulfate; AS04, Adjuvant System 04.

### 7.2 Screening and early detection

The two primary methods used for HPV screening and early detection are the Papanicolaou (Pap) smear and HPV DNA testing (125). The Pap smear assesses cellular changes suggestive of pre-cancer, while HPV DNA testing identifies high-risk HPV strains. Early identification facilitates preventative measures, underscoring the necessity for regular screening, particularly among high-risk women.

### 7.3 Safe sex practices

Safe sex, including consistent condom use and limiting sexual partners, is pivotal in reducing HPV transmission risk (126). Proper use of condoms during any sexual activity, including vaginal, anal, or oral sex, can contribute to lowering the risk of HPV transmission (127). Monogamous relationships, particularly with uninfected partners, offer substantial protection against HPV acquisition.

## 8 HPV-associated cancer management

HPV-associated cancers present a therapeutic challenge that necessitates a nuanced approach. Available interventions include surgery, radiation, and chemotherapy (128), each meticulously tailored to the cancer’s specific characteristics, including type, stage, and the patient’s health status. Surgery is a foundational treatment for HPV-associated cancers, particularly for those affecting the cervix, penis, and anus, ranging from excision of precancerous lesions to extensive tumor resections (129). Radiation therapy is integral to both primary and adjuvant treatment strategies, with a focus on targeting localized tumors or residual disease post-surgery, especially in oropharyngeal and anal cancers (130). The combination of radiation therapy with 5-FU, cisplatin, carboplatin, or mitomycin C, in single or combined regimens, significantly improves survival outcomes, independent of radiation scheduling (131). Intesity-Modulated Radiation Therapy (IMRT) has demonstrated significant advantages over conventional radiation techniques, primarily due to its ability to deliver precise radiation doses to tumours while sparing surrounding healthy tissues (132). HPV-positive HNCs have been observed to have better local control rates following radiation therapy, which is a significant factor in the overall prognosis of these patients. Studies have shown that patients with HPV-positive HNCs have improved overall survival and progression-free survival when treated with radiation therapy, compared to those with HPV-negative tumors (133). The immune response to HPV infection may play a role in the effectiveness of radiation therapy. The virus’s oncoproteins, such as E6 and E7, can disrupt the tumor’s ability to evade the immune system, potentially enhancing the therapeutic effect of radiation (134). Chemotherapy is critical for managing advanced or metastatic HPV-related cancers, often used in combination with radiation. Targeted therapies represent a paradigm shift in this therapeutic landscape, offering promising avenues for treatment (135), as illustrated by EGFR-targeted therapies in head and neck cancers.

## 9 Limitations and challenges

Global HPV vaccine coverage is suboptimal, particularly in developing regions constrained by cost and logistics. The accuracy of HPV-related cancer screenings is inconsistent, with potential for false results, and their invasive nature can impede uptake. HPV testing struggles to distinguish between transient and precancerous infections. Despite HPV’s health risks, public awareness remains low, undermining prevention. The virus’s association with other STIs complicates diagnosis and treatment, and while early-stage cancers are manageable, advanced cases necessitate less invasive and more effective therapies. There is an urgent need for research into HPV’s carcinogenic mechanisms to foster innovative treatments and preventative strategies.

## 10 Conclusion

In conclusion, significant advances have been made in combating HPV-related cancers, yet challenges in prevention, diagnosis, and treatment remain. A multidisciplinary approach is essential for progress, including vaccine accessibility, screening improvements, public education, co-infection management, and research advancement.

## Author contributions

MW: Conceptualization, Funding acquisition, Writing – original draft. HH: Writing – original draft. YT: Writing – original draft. XR: Writing – review & editing. XJ: Writing – review & editing. TM: Writing – review & editing, Funding acquisition, Validation. WL: Writing – review & editing, Funding acquisition, Supervision, Validation.
